# Regulierung von Phagenarzneimitteln: Entwicklungen, Herausforderungen und Chancen

**DOI:** 10.1007/s00103-025-04060-2

**Published:** 2025-05-05

**Authors:** Miriam Fürst-Wilmes, Vanessa Respondek, Nils Lilienthal, Katrin Buss, Anja Düchting

**Affiliations:** 1 Fachgebiet Infektiologie, Dermatologie, Allergologie, HNO, Zulassung 3, Bundesinstitut für Arzneimittel und Medizinprodukte, Bonn, Deutschland; 2 Fachgebiet Pharmazeutische Biotechnologie, Genetische und Reproduktionstoxikologie, Zulassung 2, Bundesinstitut für Arzneimittel und Medizinprodukte, Kurt-Georg-Kiesinger-Allee 3, 53175 Bonn, Deutschland

**Keywords:** Phagentherapie, Klinische Studie, Zulassung, Arzneimittel, Antibiotikaresistenz, Phage therapy, Clinical trial, Marketing authorisation, Drugs, Antibiotic resistance

## Abstract

Bakteriophagen stellen aufgrund ihrer biologischen Eigenschaften regulatorisch eine Besonderheit und gleichzeitig eine Herausforderung für die Arzneimittelzulassung dar. Etablierte europäische Leitlinien zur pharmazeutischen Qualität, Präklinik und Klinik greifen nur bedingt. Die steigende Bedrohung durch Infektionen mit multiresistenten Erregern hat nicht nur der Entwicklung von Bakteriophagen zur Behandlung von bakteriellen Infektionen in den letzten Jahren einen großen Schub verliehen, sondern auch zu größeren Fortschritten bei der Anpassung regulatorischer Anforderungen geführt. In diesem Übersichtsartikel werden diese Entwicklungen und der aktuelle Stand der regulatorischen Anforderungen an Phagenarzneimittel dargestellt.

Im Jahr 2024 wurden erstmals harmonisierte Qualitätskriterien für Phagenarzneimittel und -wirkstoffe im europäischen Arzneibuch implementiert. Sowohl die zukünftige europäische Pharmagesetzgebung als auch das nationale Medizinforschungsgesetz sollen Ausnahmeregelungen ermöglichen, die den Besonderheiten von Phagentherapeutika Rechnung tragen und neue regulatorische Gestaltungsräume öffnen. Es werden zunehmend klinische Daten zur Anwendung von Phagentherapeutika publiziert, allerdings ohne dass bisher der erhoffte Durchbruch in Form eines Wirksamkeitsnachweises über randomisierte kontrollierte klinische Studien erzielt werden konnte. Die wachsende Erfahrung mit innovativen Phagenpräparaten konnte genutzt werden, um Anpassungen der regulatorischen Anforderungen vorzunehmen. Auf dem Weg zur Zulassung eines definierten Phagenarzneimittels ist der evidenzbasierte Nachweis von Wirksamkeit und Sicherheit in randomisierten kontrollierten klinischen Studien der nächste und entscheidende Schritt.

## Abstract (en)

Due to their biological properties, bacteriophages represent a regulatory peculiarity and a challenge for drug approval. Adopted European guidelines on pharmaceutical quality, preclinical, and clinical development are only partially applicable to phage products. The increasing threat of infections with multi-resistant pathogens has not only given a major boost to the development of bacteriophages for the treatment of bacterial infections in recent years but has also led to major progress in the adaptation of regulatory requirements. This review article presents these recent developments and the current status of regulatory requirements for phage therapeutics.

In 2024, harmonised quality criteria for phage products and active pharmaceutical ingredients have been implemented in the European Pharmacopoeia for the first time. Both the EU pharmaceutical reform and the national Medical Research Act should enable exemptional regulations taking into account the special features of phage therapeutics and allow novel regulatory pathways. Although increasing clinical data on the use of phage therapeutics have been published, the desired breakthrough in the form of proof of efficacy in randomised controlled clinical trials has not yet been achieved. However, the growing knowledge on phage preparations could be used to adjust the regulatory requirements. The next and decisive step on the path to approval of a defined phage product is the evidence-based proof of efficacy and safety in a randomised controlled clinical trial.

## Einleitung

Bakteriophagen (kurz: Phagen) sind einer der Hoffnungsträger bei der Bekämpfung von Infektionen mit multiresistenten Erregern. Obwohl die ersten (dokumentierten) klinischen Anwendungen von Phagenpräparaten bald 100 Jahre zurückliegen und in Osteuropa bis heute kontinuierlich angewendet werden, fehlen etablierte Standards für die Definition der pharmazeutischen Qualität, Sicherheit und Wirksamkeit dieser Präparate [1–3]. Als Reaktion auf die größer werdende Gefahr durch multiresistente Bakterien wurde in den letzten Jahren eine Vielzahl von Förderinitiativen, auch in Deutschland, gestartet, die der Entwicklung von Bakteriophagen als Arzneimittel (AM) zu neuem Auftrieb verholfen haben [4]. Eine der Herausforderungen blieb dabei, die Phagen-Arzneimittel (Phagen-AM) mit ihren besonderen biologischen Eigenschaften und Wirkungsweisen in den bestehenden regulatorischen Rahmen einzuordnen bzw. diesen zu adaptieren. Vorgaben an die Qualität, Sicherheit und Wirksamkeit von Phagenpräparaten haben sich in den letzten Jahren in Deutschland wie auch in Europa entwickelt. Dieser Artikel soll eine Übersicht über diese Entwicklungen geben und den aktuellen Stand der regulatorischen Anforderungen an Phagen-AM abbilden.

## Qualitätsaspekte

### Herstellung und Anwendung von Phagenarzneimitteln.

In Europa werden Bakteriophagen zur therapeutischen Anwendung am Menschen als sogenannte biologische Arzneimittel im Sinne von Art. 1 Nr. 2, 3 in Verbindung mit Anhang I, Teil 1 3.2.1.1 lit. b) Sätze 3 und 4 Richtlinie 2001/83/EG betrachtet.^1^ In Deutschland gelten sie als Funktionsarzneimittel gemäß § 2 Abs. 1 in Verbindung mit § 3 Nr. 4 Arzneimittelgesetz (AMG). Sie bedürfen grundsätzlich vor der Anwendung am Patienten entweder einer arzneimittelrechtlichen Marktzulassung oder einer Genehmigung zur Verwendung als Prüfpräparate in einer klinischen Studie.

Phagen-AM werden aktuell über 2 unterschiedliche Wege hergestellt und angewandt:

1. Standardisierte Phagenpräparate (oft Mischungen verschiedener Phagen) werden im Voraus industriell produziert und im Rahmen einer klinischen Prüfung eingesetzt. Diese AM unterliegen der Zulassungs- und Herstellungserlaubnispflicht und können prinzipiell entsprechend den bereits bestehenden regulatorischen Strukturen über klinische Studien auf ihre Qualität, Wirksamkeit und Sicherheit überprüft und schlussendlich zugelassen werden. Als zugelassene AM stünden sie allen Patienten entsprechend der genehmigten Indikation zur Verfügung. Aktuelle regulatorische Vorgaben z. B. für Änderungen der Zusammensetzung von zugelassenen Phagen-AM stellen für diese Produkte jedoch Herausforderungen dar (z. B. Austausch/Anpassung der Phagen im AM durch Resistenzentwicklung der bakteriellen Infektionserreger – „moving targets“). Die künftige europäische Pharma-Legislative könnte hier passendere Strukturen und regulatorische Konzepte etablieren.
2. Phagenpräparate werden als individuelle Rezeptur-AM nach Verschreibung eines Arztes meist in einer (Krankenhaus‑)Apotheke zeitnah vor der Anwendung spezifisch für den einzelnen Patienten hergestellt. Für diese Produkte (*Formula magistralis*) bestehen Ausnahmen von der Zulassungs- und Genehmigungspflicht, da sie als solche gemäß Art. 3 Abs. 1 der RL 2001/83/EG nicht in den Geltungsbereich der Richtlinie fallen. In Deutschland besteht die Zulassungspflicht nach § 21 Abs. 1 AMG für Fertigarzneimittel (definiert in § 4 Abs. 1. S. 1 AMG), nicht jedoch für individuelle Rezepturarzneimittel, da diese im Sinne der Definition des Fertigarzneimittels in § 4 Abs. 1 Nr. 1 AMG nicht „im Voraus“, sondern im Sinne von § 1a Abs. 8 APBetrO „in der Apotheke im Einzelfall auf Grund einer Verschreibung oder auf sonstige Anforderung einer einzelnen Person … hergestellt“ werden. Die Anwendung erfolgt meist im individuellen Heilversuch (vgl. § 37 der Deklaration von Helsinki). Änderungen der zulassungsfreien Phagen-AM können flexibler gehandhabt und schneller umgesetzt werden, da regulatorische Schritte zur Genehmigung der Änderungen entfallen. Allerdings steht diese „Last-resort“-Behandlung nur einer geringen Anzahl an Patienten in Ausnahmesituationen zur Verfügung.

### Qualität der Phagen-AM.

Gemäß § 55 Abs. 8 AMG müssen alle Arzneimittel in Übereinstimmung mit den anerkannten pharmazeutischen Regeln hergestellt werden. Diese Regeln sind entsprechend § 55 Abs. 1 AMG im Arzneibuch niedergelegt. Sie umfassen u. a. die Qualität von Arzneimitteln und den bei ihrer Herstellung verwendeten Stoffen. Durch die Regeln des Arzneibuchs wird ein allgemeiner Mindeststandard an die Qualität von Arzneimitteln und deren Inhaltsstoffen festgelegt, um damit die Sicherheit für den Patienten zu gewährleisten. Im März 2024 wurde auf der 178. Sitzung der Europäischen Arzneibuchkommission das allgemeine Kapitel „Phage therapy medicinal products (5.31)“ verabschiedet. Die Veröffentlichung erfolgte im Juli 2024 im Supplement 11.6 des Europäischen Arzneibuchs (Ph.Eur.); es trat im Januar 2025 in Kraft. Das Kapitel wird rechtlich verbindlich, wenn es in einer Monografie referenziert wird (oder es in einem anderen allgemeinen Kapitel referenziert wird, das wiederum in einer Monografie referenziert wird). In diesem Kapitel werden erstmals europaweit harmonisierte Qualitätskriterien für humane Phagen-AM und -wirkstoffe definiert. Dabei umfasst es zulassungsfreie wie auch zulassungspflichtige Human- und Tierarzneimittel. Um diesem breiten Spektrum an sehr unterschiedlichen Produkten gerecht zu werden, etabliert das Kapitel grundlegende Anforderungen, wobei eine ausreichende Flexibilität ermöglicht wird.

Die Qualitätskriterien beinhalten u. a. Vorgaben zu:

- Bakterien- und Phagenbänken. inkl. Qualitätskriterien (u. a. Identität, mikrobielle Reinheit, Aktivität),
- Produktion und Aufreinigung, inkl. In-Prozess-Kontrollen,
- Wirkstoff- und Fertigproduktspezifikationen, inkl. relevanter Qualitätskriterien (u. a. Identität, mikrobielle Qualität, Aktivität).

Ein zentrales Qualitätskriterium aller Phagen-AM stellt die biologische Aktivität dar, welche meist anhand eines „Plaque-Assays“ bestimmt wird; diese Methode ist jedoch bisher nicht standardisiert. Auf der 176. Sitzung der Europäischen Arzneibuchkommission im Juni 2023 wurde beschlossen, ein neues allgemeines Kapitel (2.7.38) zur Bakteriophagen-Aktivitätsbestimmung zu verfassen, um damit harmonisierte Angaben zur Durchführung des Assays sowie seiner Validierung zu geben. Eine Veröffentlichung des Kapitelentwurfs zur Kommentierung erfolgte im April 2025.

Um die Anforderungen an zulassungspflichtige Phagen-AM darzustellen, thematisiert die Europäische Arzneimittelagentur (EMA) diese in spezifischen „Guidelines“. Für Tierarzneimittel wurde die „Guideline on quality, safety and efficacy of veterinary medicinal products specifically designed for phage therapy“, (EMA/CVMP/NTWP/32862/2022) veröffentlicht, während für humane Phagentherapeutika eine entsprechende Guideline aktuell erarbeitet wird („Guideline on the development and manufacture of human medicinal products specifically designed for phage therapy“, EMA/CHMP/BWP/1/2024). Relevante Aspekte (auch unter Berücksichtigung des Kapitels Ph.Eur. 5.31) der letztgenannten Guideline sind u. a.:

- die Qualität der Ausgangs‑, Hilfs- und Wirkstoffe,
- die Herstellung und Spezifikationen der Bakterien- und Phagenbänke,
- die Charakterisierung des Wirkstoffes und seiner Verunreinigungen (z. B. Endotoxine),
- das Herstellungsverfahren, inkl. Prozessentwicklung, -validierung und -kontrollen,
- die Wirkstoff- und Fertigproduktspezifikationen, inkl. analytischer Methoden und Validierung,
- Referenzstandards und Stabilität.

Eine Veröffentlichung des Guideline-Entwurfs zur Kommentierung ist für Mitte 2025 geplant. Darüber hinaus sind grundsätzlich auch für Phagen-AM die Prinzipien der bestehenden EMA- und ICH-Leitlinien für Biotech-Produkte – soweit zutreffend – anwendbar, z. B. hinsichtlich Charakterisierung von Zellbänken (ICH Q5D), Stabilität (ICH Q5C), Spezifikationen (ICH Q6B), analytischer Methoden (ICH Q2, Q14) sowie weiterer Themen (ICH Q8–Q11). Relevant für Phagenprodukte als klinische Prüfpräparate ist insbesondere die „Guideline on the requirements for quality documentation concerning biological investigational medicinal products in clinical trials“ (EMA/CHMP/BWP/534898/2008 Rev. 2).

Aktuell wird auch von der Arbeitsgemeinschaft der Wissenschaftlichen Medizinischen Fachgesellschaften (AWMF) eine S2k-Leitlinie erarbeitet; die Finalisierung ist für Juli 2025 geplant. Die Leitlinie wird Empfehlungen hinsichtlich der personalisierten Bakteriophagenherstellung und -therapie beinhalten und dabei auf rechtliche und logistische Aspekte in Deutschland eingehen. Durch den Konsensusprozess und die Beteiligung der verschiedenen Fachgesellschaften ergibt sich eine harmonisierte Sammlung von Handlungsempfehlungen unterschiedlicher Evidenzgrade, die Anleitung und Rahmenbedingungen u. a. zur Herstellung von personalisierten Phagen-AM und deren Qualitätsparametern und -kontrollen geben kann.

Die Vielzahl der Aktivitäten auf dem Gebiet der Qualität und Herstellung von Phagen-AM unterstreicht das mögliche Potenzial dieser Produkte, auch wenn der klinische Wirksamkeitsnachweis aus randomisierten kontrollierten klinischen Studien weiterhin aussteht. Durch systematisierte Einzelfallbeobachtungen scheint deutlich zu werden, dass die Phagentherapie (in Kombination mit Antibiotika) eine valide Option zur Behandlung von Infektionen durch antibiotikaresistente Erreger darstellen kann [5]. Über die zuvor beschriebenen Novellierungen wurden/werden nun spezifische Vorgaben für diese besondere AM-Gruppe festgelegt, die eine europaweit harmonisierte und konsistent hohe Qualität dieser Produkte auch im Sinne des Patientenschutzes gewährleisten sollen.

### Herstellungserlaubnis und Gute Herstellungspraxis.

Grundsätzlich ist in Europa die Herstellung von gewerblich zubereiteten AM erlaubnispflichtig (Art. 40 RL 2001/83/EG, § 13 Abs. 1 AMG) und erfordert die Einhaltung der Grundsätze und Leitlinien der Guten Herstellungspraxis (GMP), was auch im Rahmen der Erteilung einer Herstellungserlaubnis (HE) überprüft wird (§ 1 Abs. 1 i. V. m § 2 Abs. 3 und § 13 Abs. 1 Arzneimittel- und Wirkstoffherstellungsverordnung – AMWHV).

Die personalisierte Herstellung von AM in einer Apotheke im Rahmen des üblichen Apothekenbetriebs nach ärztlicher Verschreibung für einen bestimmten Patienten unterliegt nicht der Erlaubnispflicht (§ 13 Abs. 2 S. 1 AMG), jedoch muss auch diese Herstellung in Übereinstimmung mit den anerkannten pharmazeutischen Regeln erfolgen (§ 55 Abs. 8 AMG) und die Qualität der Ausgangsstoffe und des fertigen AM muss gesichert sein (§ 6 Abs. 1 i. V. m. § 11 ApBetrO). Die Entscheidung über die Einordnung der Herstellung individualisierter Phagen-AM als „üblicher Apothekenbetrieb“ erfolgt durch die zuständige Überwachungsbehörde. Sollte die Einordnung als „üblicher Apothekenbetrieb“ nicht erfolgen, so ist die Herstellung gemäß § 13 Abs. 1 AMG erlaubnispflichtig.

Eine personalisierte Herstellung von Phagen-AM kann prinzipiell auch durch Ärzte („unter unmittelbarer fachlicher Verantwortung zum Zwecke der persönlichen Anwendung bei einem bestimmten Patienten“) erfolgen. Bei Verwendung eines GMP-konformen Phagenwirkstoffes ist diese Tätigkeit erlaubnisfrei nach § 13 Abs. 2b AMG (wenn auch anzeigepflichtig nach § 67 Abs. 2 AMG). Eine Phagenwirkstoffherstellung durch einen Arzt zur Herstellung eines Phagen-AM unterliegt jedoch der Erlaubnispflicht nach § 13 Abs. 1 S. 3 AMG.

Die Herstellung von gentechnisch veränderten oder rekombinant hergestellten Phagen-AM (definiert als Arzneimittel für neuartige Therapien – ATMP) unterliegt in jedem Fall der Erlaubnispflicht (§ 13 Abs. 1 AMG).

Mit dem Medizinforschungsgesetz, das das AMG und die AMWHV geändert hat, wird den Besonderheiten der individualisierten Phagentherapeutika Rechnung getragen. Die Bundesoberbehörden werden mit dem neu eingeführten § 14 Abs. 6 AMG ermächtigt, „für Arzneimittel, die als individuelle Zubereitung für einen einzelnen Patienten hergestellt und unter der fachlichen Verantwortung eines Arztes zur antibakteriellen Therapie angewendet werden, Empfehlungen zur Auslegung der Grundsätze und Leitlinien der Guten Herstellungspraxis zu veröffentlichen“. Auch kann nach dem neuen § 14 Abs. 7 AMG „die zuständige Bundesoberbehörde auf Antrag einer zuständigen Behörde eine Stellungnahme zur Auslegung der Grundsätze und Leitlinien der Guten Herstellungspraxis für die zuvor genannten AM … erstellen und veröffentlichen“. Die AMWHV wurde entsprechend in § 3 Abs. 2 mit Verweis auf den § 14 Abs. 6 AMG ergänzt.

Diese Neuerungen ermöglichen individuell und risikobasiert die allgemeinen GMP-Vorgaben spezifisch an die Phagen-AM-Herstellung anzupassen und dabei eine gleichbleibend hohe Qualität und Sicherheit dieser Produkte zu gewährleisten. Die GMP-Vorgaben können damit stärker die besonderen Eigenschaften der Phagen als AM berücksichtigen. Eine Veröffentlichung der Empfehlungen nach § 14 Abs. 6 AMG ist für Mitte 2025 geplant. Die Empfehlungen sind für die Überwachungsbehörden rechtlich nicht bindend, jedoch sollte ein Abweichen davon begründet werden.

### Ausblick zur Regulation von Phagenarzneimitteln.

Die aktuelle regulatorische Umgebung kann den besonderen Anforderungen der Phagen-AM nicht vollständig entsprechen. Herausforderungen bestehen hinsichtlich der Zulassung bzw. nachfolgender Änderung des Phagen-AM, um neue Phagen(stämme) bei Resistenzentwicklung einzuführen oder auszutauschen, ohne dass für jeden (neuen) Phagen eine einzelne Zulassung beantragt werden müsste. Die Nutzung einer „Plattform“-Technologie und die Möglichkeit einer Zulassung von Phagen-AM mit (teil-)variabler Zusammensetzung wären hier ggf. hilfreich, um die Herstellung (inkl. Erlaubnis) und Zulassung verschiedener Phagen(stämme) regulatorisch zu bündeln.

In den Vorschlägen der Europäischen Kommission für eine neue Arzneimittelrichtlinie als „Nachfolgerin“ der Richtlinie 2001/83/EG und eine neue Arzneimittelverordnung als „Nachfolgerin“ der Verordnung (EG) Nr. 726/2004, (vgl. Dokumente 2023/0132 (COD) und 2023/0131 (COD), nachfolgend: Vorschläge für eine neue EU-Richtlinie bzw. -Verordnung) werden (zulassungspflichtige) Phagen-AM und die zuvor beschriebenen Herausforderungen berücksichtigt [6]. Hier werden 2 neue Konzepte eingeführt: „adapted frameworks“ (Artikel 28 des Vorschlags für eine neue EU-Richtlinie) und „regulatory sandbox“ (Artikel 113–115 des Vorschlags für eine neue EU-Verordnung). Diese beinhalten „angepasste, erweiterte, ausgesetzte oder aufgeschobene Anforderungen“. Weiterhin werden neue Zulassungsmöglichkeiten für AM geschaffen, „die variable und feste Bestandteile enthalten, um damit z. B. verschiedene Varianten eines Infektionserregers zu bekämpfen oder … anzupassen (‚Plattformtechnologie‘)“ (Artikel 15 des Vorschlags für eine neue EU-Richtlinie). Diese neuen und flexiblen Wege in der Regulation könnten sich für Phagen-AM als sehr hilfreich erweisen, um sowohl Zulassungen als auch spätere Änderungen der Produkte pragmatisch zu handhaben und damit Phagen-AM für alle Patienten als Therapieoption verfügbar zu machen.

## Präklinische Aspekte

Im Folgenden werden die den Überlegungen zum präklinischen Entwicklungsprogramm zugrunde liegenden Annahmen dargelegt: Bakteriophagen sind Biologika, die sich nicht in eukaryotischen Zellen vermehren und dort keine direkten pharmakologischen Wirkungen aufweisen. Daher gelten virulente Phagen als ungefährlich (atoxisch) für den Menschen. Sowohl Tiere als auch Menschen sind ständig auf natürliche Weise großen Mengen an Bakteriophagen ausgesetzt [7, 8].

Abschnitte der „Guideline on the evaluation of medicinal products indicated for treatment of bacterial infections“ (CPMP/EWP/558/95 Rev. 3) lassen sich auf Phagen-AM (Abschn. 4.1 Nicht-klinische Bewertung der antibakteriellen Aktivität) übertragen und die Leitlinie für veterinäre Phagenprodukte (EMA/CVMP/NTWP/32862/2022) enthält ebenfalls Hinweise für das präklinische Programm. Während bislang keine spezifische Leitlinie für die Phagen-AM und infolgedessen auch keine Beschreibung der Anforderungen an die Präklinik im Bereich der humanen Arzneimittelentwicklung existieren, bieten die oben genannten Leitlinien eine erste hilfreiche Orientierung für regulatorische Aspekte. Es ist allgemein anerkannt, dass regulatorische Anforderungen für neue Arzneimittel in einem angemessenen Verhältnis zu den mit ihrer beabsichtigten Anwendung verbundenen Risiken stehen sollten. Darauf basierend können Abweichungen bzw. Reduzierungen vom standardisierten präklinischen Prüfumfang einer Arzneimittelentwicklung zur Prüfung des Wirkstoffs – *Pharmakologie, Pharmakodynamik, Pharmakokinetik (PK), Toxizität nach wiederholter Gabe, Prüfung auf Genotoxizität, Kanzerogenität und Reproduktionstoxizität *und weitere Toxikologieendpunkte wie *lokale Toleranz *– wissenschaftlich begründet werden. Dennoch sollte der Antragsteller die Unbedenklichkeit und Sicherheit der ausgewählten Phagen im Hinblick auf die Toxizität diskutieren und das nicht-klinische Dossier bei einem Zulassungsantrag durch entsprechende Informationen und Diskussionen unterstützen.

Potenzielle Sicherheitsbedenken ergeben sich aus der biologischen Wirkung von Phagen: Das Abtöten von Bakterien kann zu einer Freisetzung von Endotoxinen und anderen hochwirksam entzündungsfördernden Substanzen führen [9]. Endotoxine und Exotoxine als mögliche mikrobiologische Kontamination sind wichtige Qualitätskriterien des Produktes. Diese und weitere bakterielle Komponenten müssen in Phagenprodukten kontrolliert werden. Die bisherigen Erfahrungen in diesem Gebiet zeigen, dass die Produktqualität (Reinheit) ein wesentlicher Faktor für die Sicherheit ist (siehe Abschnitt Qualitätsaspekte).

*In-vitro*-Empfindlichkeitsstudien sollten die Wirksamkeit der Phagen auf die bakteriellen Infektionserreger nachweisen. Die Studien sollten unter Bedingungen durchgeführt werden, welche die Situation am Infektionsort (z. B. Vorhandensein eines Biofilms) bestmöglich widerspiegeln. Bei der Verwendung von Kombinationen mehrerer Phagen ist sicherzustellen, dass diese für ein repräsentatives klinisches Isolat miteinander kompatibel sind. Auch wenn die gängigen Studien zu Absorption, Verteilung, Metabolismus und Ausscheidung (PK) für Phagenprodukte als ungeeignet angesehen werden, sollte der Antragsteller die Absorption am Ort der Verabreichung und Verteilung sowie die zu erwartenden Abbauwege der Phagen untersuchen oder entsprechende Informationen auch auf Basis von Literaturdaten diskutieren. In Abhängigkeit von der angestrebten Applikationsart (z. B. inhalativ oder intravenös) sind unterschiedliche PK-Daten erforderlich.

Aufgrund von Antibiotikaresistenzen werden Antibiotika bereits seit Längerem in Kombination mit Phagen untersucht. Antagonistische oder synergistische Wechselwirkungen zwischen Phagen und Antibiotika sollten bei der Entwicklung eines Phagen-AM berücksichtigt werden. Es wurde berichtet, dass das Resultat der Wechselwirkungen zwischen Phagen und Antibiotika von verschiedenen Faktoren abhängig ist, u. a. von der Antibiotikaklasse, Art der Phagen und Stöchiometrie der Paarung [10]. Des Weiteren können auch für die Phagentherapie und/oder „Phagen‑/Antibiotika-Kombinationstherapie“ Resistenzmechanismen und eine phageninduzierte Immunantwort von Relevanz sein [11, 12]. Diese Aspekte sollten ebenfalls diskutiert oder in den Anfängen der Entwicklung untersucht werden.

Hinsichtlich der genannten Aspekte besteht aktuell keinerlei Vorgabe, dass *In-vivo*-Tierstudien durchgeführt werden müssen. Vielmehr obliegt es dem Antragsteller, die Effektivität der ausgewählten Phagen gegen die Zielbakterien nachzuweisen, die Unbedenklichkeit des Phagenprodukts zu belegen sowie die sichere und effektive Dosis beim Menschen zu ermitteln.

Das Bundesinstitut für Arzneimittel und Medizinprodukte (BfArM) als zuständige Zulassungsbehörde (bei nicht gentechnisch veränderten Phagen) erwartet für Phagen-AM keine *Stand-alone*-Studien zur Reproduktions- und Entwicklungstoxikologie. Es wird angenommen, dass virulente Bakteriophagen nicht direkt mit der DNA oder anderem chromosomalen Material interagieren. Somit kann ebenfalls auf die Standardbatterie von Genotoxizitätstests und auf Kanzerogenitätsstudien verzichtet werden.

Abschließend ist festzuhalten, dass ein geplantes, begründet reduziertes und gezieltes präklinisches Programm für Phagen-AM den regulatorischen Anforderungen für eine sichere Arzneimittelentwicklung gerecht werden kann.

## Klinische Aspekte

Phagen werden bereits seit mehr als 100 Jahren angewendet und trotzdem steht der evidenzbasierte Nachweis der therapeutischen Wirksamkeit der Phagentherapie basierend auf einer angemessen großen und gut geplanten randomisierten kontrollierten klinischen Studie („randomised controlled clinical trial“ – RCT), die Voraussetzung für die Marktzulassung eines Phagen-AM ist, noch aus. Aus regulatorischer Sicht sind Wirksamkeitsdaten aus Phase-2- und Phase-3-Studien dringend erforderlich, um die Phagentherapie als wirksame Behandlungsoption anzuerkennen und regulatorische Anforderungen anzupassen. Daten zur Wirksamkeit und Sicherheit von Phagen stammen hauptsächlich aus Einzelanwendungen und unkontrollierten Studien. In den wenigen durchgeführten und publizierten RCTs (nur Phase-1/2- und Phase-2-Studien; Tab. 1) konnte die Wirksamkeit der Phagentherapie in den meisten Fällen nicht ausreichend belegt werden [13–15].

**Tab. 1:** Beispiele publizierter randomisierter kontrollierter klinischer Studien (Phase 1/2 und 2), in denen u. a. die Wirksamkeit von Phagen untersucht wurde (Stand 02/2025). Die Ergebnisse dieser Studien (negativ und positiv) sollten bei der Planung weiterer Studien (Phase 2 und Phase 3) berücksichtigt werden, um den noch ausstehenden Wirksamkeitsnachweis der Phagentherapie zu erbringen

Studienphase	Studienbeschreibung	Eingeschlossene Patienten	Phagenprodukt; Anwendung; Dosierung	Ergebnis in Bezug auf die Wirksamkeit	Referenz
Phase 1/2	Randomisierte, placebokontrollierte, doppelblinde Studie in erwachsenen Patienten mit chronischer Otitis media durch *Pseudomonas aeruginosa*	*n* = 24	Fixer Cocktail aus 6 Phagen; topische Anwendung; Einmalgabe von 6 × 10^5^ PFU	Signifikante klinische Verbesserung	[19]
				Reduzierung der Bakterienzahl	
				Replikationsnachweis der angewendeten Phagen	
	Randomisierte, placebokontrollierte Studie in hospitalisierten Kindern (6–24 Monate) mit akuter bakterieller Diarrhö durch *Escherichia coli*	*n* = 120	Fixer Cocktail aus 11 Phagen oder kommerzielles Produkt aus Russland (aus mind. 17 Phagen); orale Anwendung; 3,6 × 10^8^ PFU bzw. 1,4 × 10^9^ PFU über 4 Tage	Keine klinische Verbesserung	[13]
				Keine Replikation der Phagen im Darm	
				*Gründe für negatives Ergebnis:*	
				Nur 60 % der eingeschlossenen Patienten hatten eine nachgewiesene *E.-coli*-Infektion	
				Unzureichende Abdeckung des Phagencocktails	
				Geringerer *E.-coli*-Titer als erwartet	
				pH-Stabilität des Phagencocktails unklar	
	Randomisierte, placebokontrollierte, doppelblinde, multizentrische Studie in erwachsenen Patienten mit *Pseudomonas-aeruginosa-*infizierten Brandwunden (Studie „PhagoBurn“)	*n* = 27	Fixer Cocktail aus 12 Phagen; topische Anwendung; 1 × 10^6^ PFU/ml für 7 Tage	Studie wurde wegen mangelnder Rekrutierung und mangelnder Wirksamkeit vorzeitig abgebrochen	[14]
				*Gründe für negatives Ergebnis*:	
				Instabilität des Phagencocktails und dadurch zu geringe angewendete Phagenkonzentration	
Phase 2	Randomisierte, placebokontrollierte, doppelblinde Studie zur Behandlung von Harnwegsinfektionen (durch *Enterococcus* spp., *E. coli, Proteus mirabilis, P. aeruginosa, Staphylococcus* spp., *Streptococcus* spp.) in Männern mit geplanter transurethraler Resektion der Prostata	*n* = 113	Kommerzieller Phagencocktail aus Georgien; topische Anwendung (intravesikal); 2 × täglich für 7 Tage	Kein Wirksamkeitsunterschied zwischen den Behandlungsarmen	[15]
				*Gründe für negatives Ergebnis:*	
				Zu breite Indikation	
				Hohe Spontanheilungsrate bei unkomplizierten Harnwegsinfektionen	

In einer kürzlich veröffentlichten retrospektiven Beobachtungsstudie von Pirnay et al. [5] wurde die Wirksamkeit von 100 Einzelanwendungen mit einer Wirksamkeitsrate (klinischen Verbesserung) von 77 % beschrieben. Die Aussagekraft dieser Daten ist jedoch limitiert, da es keine Kontrollgruppe gab und die Wirksamkeit (und Sicherheit) der Phagenanwendung nicht auf der Grundlage von vordefinierten Kriterien beurteilt wurde. Darüber hinaus wurden die Phagen für unterschiedliche Indikationen (in 70 % der Fälle in Kombination mit einem Antibiotikum angewendet) und auf unterschiedliche Weise appliziert. Trotzdem kann die Erfahrung aus diesen und anderen personalisierten Einzelanwendungen in die Planung von RCTs eingebracht werden (z. B. im Hinblick auf die Dosierung und die Anwendungsart und -dauer).

Zum aktuellen Zeitpunkt existieren keine festen Vorgaben zum klinischen Entwicklungsprogramm für Phagen-AM für die humane Anwendung. Die „Guideline on the evaluation of medicinal products indicated for treatment of bacterial infections“ (CPMP/EWP/558/95 Rev 3) ist jedoch größtenteils für humane Phagenprodukte anwendbar (z. B. Abschn. 5: „General considerations for clinical programmes“).

Basierend auf Erfahrungen aus bereits publizierten RCTs und wissenschaftlich-regulatorischen Beratungen auf nationaler und EU-Ebene sollten aus regulatorischer Sicht die folgenden Aspekte für die Planung von Phase-2- und Phase-3-Studien und den Wirksamkeitsnachweis von Phagen-AM berücksichtigt werden:

### Anwendungsgebiet.

Die Anwendung eines Phagenpräparates ist sowohl für die Therapie bakterieller Infektionen als auch als Prophylaxe durch die Eradikation eines potenziellen Krankheitserregers in einer bestimmten Körperregion denkbar. Die hohe Wirtsspezifität von Phagen schließt jedoch eine breite Indikation im Sinne herkömmlicher Antibiotika aus. Für eine Proof-of-Concept(PoC)-Studie (Phase 2) eignen sich insbesondere (akute) Infektionen, die ausschließlich oder zu einem sehr großen Anteil nur durch eine Bakterienart ausgelöst werden (monobakterielle Infektionen) und für die eine lokale Applikation der Phagen möglich ist.

Das Studiendesign einer pivotalen Studie (Phase 3) richtet sich vor allem danach, ob das Phagenprodukt allein oder in Kombination mit Antibiotika angewendet werden soll. Insbesondere bei schweren und/oder chronischen Infektionen könnte eine Kombinationstherapie aus Phagen und Antibiotika sinnvoll sein. In diesem Fall muss eine Überlegenheit der Kombinationstherapie gegenüber dem Therapiestandard gezeigt werden. Bei weniger schwerwiegenden Infektionen sollte für das Phagenpräparat Nichtunterlegenheit gegenüber der Standardantibiotikatherapie (oder Placebo, falls kein Therapiestandard existiert) nachgewiesen werden.

### Dosierung und Art der Anwendung.

Für eine erfolgreiche Therapie ist es erforderlich, dass eine ausreichend hohe Anzahl an Phagen an den Infektionsort gelangt. Dabei wird insbesondere eine Vermehrung der Phagen mit anschließender Lyse der Zielbakterien („aktive Therapie“) angestrebt. Die Pharmakokinetik der Phagen ist jedoch noch unzureichend verstanden und die Frage, ob eine hohe Konzentration immer mit einem besseren Therapieerfolg einhergeht, ist noch nicht abschließend geklärt. Zusätzlich hängt die Vermehrung der Phagen von der Anzahl der Bakterien am Infektionsort ab [5, 16, 17].

Nichtsdestotrotz können mit einer lokalen Applikation (z. B. topisch oder inhalativ) höhere Phagenkonzentrationen am Infektionsort erreicht werden. Erfahrungen aus Einzelanwendungen bzgl. der eingesetzten Phagenkonzentration, des Dosierungsintervalls und der Therapiedauer sollten in die Planung von RCTs einfließen. Eine Überprüfung der Phagenreplikation am Infektionsort im Rahmen von RCTs wird empfohlen.

### Diagnostik.

Phagen sind im Gegensatz zu den meisten Antibiotika sehr spezifisch für eine Bakterienart, sodass der Infektionserreger bereits vor Beginn der Phagentherapie identifiziert sein muss. Eine differenzierte mikrobielle Diagnostik ist daher auch im Hinblick auf den Therapieerfolg zwingend erforderlich.

Eine Empfindlichkeitstestung ist ratsam, aber nicht zwingend vor Einschluss der Patienten in die Studie erforderlich (z. B. bei einem fixen Phagencocktail mit einer hohen Abdeckungsrate). Die Empfindlichkeit des Bakterienisolats gegenüber einzelnen Phagen oder dem Phagencocktail sollte jedoch im Rahmen einer klinischen Studie unbedingt getestet werden, um die Ergebnisse des Empfindlichkeitstests mit der Wirksamkeit der Therapie korrelieren zu können. In einer pivotalen Studie sollte die primäre Analyse basierend auf allen Patienten mit phagenempfindlichen Bakterien beruhen („microbiological-ITT population“; (CPMP/EWP/558/95 Rev 3)). Wiederholte Empfindlichkeitstestungen während der Therapie können Hinweise auf eine mögliche Resistenzentwicklung der Bakterien geben und je nach Studiendesign auch zu einer Anpassung der Therapie führen.

### Endpunkte.

Für eine PoC-Studie kann ein primärer mikrobiologischer Endpunkt (z. B. Reduzierung der Bakterienlast) als Surrogatparameter für die Wirksamkeit gewählt werden. Es wird jedoch im Hinblick auf die weitere Entwicklung empfohlen, bereits in einer PoC-Studie klinische Endpunkte zu untersuchen, die Rückschlüsse auf den klinischen Nutzen der Phagentherapie ermöglichen. Relevante klinische Endpunkte könnten z. B. Zeit bis zur klinischen Verbesserung der Symptomatik, Dauer der Hospitalisierung, Notwendigkeit (oder Dauer) einer zusätzlichen Antibiotikatherapie, Häufigkeit von Reinfektionen oder die Infektionsrate bei einer prophylaktischen Anwendung sein.

### Patientenpopulation.

Im Einklang mit der geplanten Indikation sollte die in eine Studie eingeschlossene Patientenpopulation klar definiert werden. Dabei sollte z. B. die Häufigkeit eines bestimmten Erregers in einer bestimmten Indikation berücksichtigt werden, um eine ausreichend große Anzahl an Patienten rekrutieren zu können. Des Weiteren sollte unter Berücksichtigung der möglichen Risiken einer Phagentherapie abgewogen werden, welche Patienten am meisten von der Phagentherapie profitieren könnten und in welchen Patienten mit hoher Wahrscheinlichkeit ein Effekt gezeigt werden kann (Nutzen-Risiko-Analyse).

Generell wurde in Bezug auf die Sicherheit bei der Anwendung von Phagen im Rahmen von klinischen Studien [13, 14, 18, 19] als auch in individuellen Heilversuchen [5, 20] bisher ein gutes Sicherheitsprofil beschrieben. In einem 2022 veröffentlichten systematischen Review von 52 Studien zu Sicherheit und Wirksamkeit der Phagentherapie bei schwer zu behandelnden Infektionen wurden Nebenwirkungen in 33 (7 %) von 441 Patienten, die sich einer Phagentherapie unterzogen, und bei 37 (15 %) von 249 Patienten in der Kontrollgruppe berichtet [20]. Im Allgemeinen waren diese unerwünschten Ereignisse in ihrer Ausprägung mild und verschwanden nach Beendigung der Phagenbehandlung. Die gute Verträglichkeit der Phagentherapie im Hinblick auf das Nutzen-Risiko-Verhältnis könnte daher einen Vorteil gegenüber der Antibiotikatherapie, insbesondere solcher mit schweren Nebenwirkungen, bieten. Langfristig wäre auch eine systematische Untersuchung der Wirksamkeit in besonderen Patientenpopulationen, wie z. B. Kindern und Jugendlichen oder immunsupprimierten Patienten, erstrebenswert.

### Weitere Aspekte.

Die Neutralisierung von Phagen durch Antikörper wurde bereits in der Literatur beschrieben und hängt vermutlich von der Therapiedauer, dem Immunstatus des Patienten und der Art der Anwendung (z. B. intravenöse Gabe) ab [5, 17, 21]. Weitere Daten zu neutralisierenden Antikörpern sowie die Korrelation von Antikörpernachweis und Wirksamkeit der Therapie sind wünschenswert und sollten im Rahmen von klinischen Studien systematisch erhoben werden. Zur Erhöhung/Erhaltung der therapeutischen Wirksamkeit sind auch Anpassungen der Phagen im AM auf das bakterielle Patientenisolat denkbar, z. B. in Form von „Phagentraining“ oder durch gentechnische/synthetische Modifikation der Phagen [22, 23].

## Fazit

Hinsichtlich der Regulierung von Phagentherapeutika hat es in der letzten Zeit verschiedene Neuerungen gegeben. Die Veröffentlichung von Richtlinien für europaweit harmonisierte Qualitätskriterien für Phagen-AM und die Anpassung der nationalen und europäischen Pharmagesetzgebung an die Besonderheiten von Phagentherapeutika zeigen, wie viel Bewegung derzeit in diesem Feld ist und dass wichtige Hürden genommen wurden. Das Ziel der Zulassung des ersten Phagen-AM ist jedoch noch nicht erreicht. Hierfür wird entscheidend sein, dass aus den Fehlern bisher durchgeführter klinischer Studien gelernt wird, um gute klinische Studien zu planen, die die Wirksamkeit und Sicherheit von definierten Phagenarzneimitteln nachweisen können. Eine enge Abstimmung mit den regulatorischen Behörden in Form von wissenschaftlicher Beratung kann dabei helfen, Fehler im gesamten Entwicklungsprogramm zu vermeiden.

## Footnotes

1 Eine abweichende Ansicht wird im Artikel von Faltus in diesem Themenheft dargestellt.
